# Cognitive and mental health changes and their vulnerability factors related to COVID-19 lockdown in Italy

**DOI:** 10.1371/journal.pone.0246204

**Published:** 2021-01-27

**Authors:** Eleonora Fiorenzato, Silvia Zabberoni, Alberto Costa, Giorgia Cona

**Affiliations:** 1 Department of General Psychology, University of Padua, Padua, Italy; 2 IRCCS Fondazione Santa Lucia, Rome, Italy; 3 Niccolò Cusano University, Rome, Italy; 4 Padova Neuroscience Center, University of Padua, Padua, Italy; Nathan S Kline Institute, UNITED STATES

## Abstract

The COVID-19 pandemic and government imposed social restrictions like lockdown exposed most individuals to an unprecedented stress, increasing mental health disorders worldwide.

We explored subjective cognitive functioning and mental health changes and their possible interplay related to COVID-19-lockdown. We also investigated potential risk factors to identify more vulnerable groups. Across Italy, 1215 respondents completed our Qualtrics-based online-survey during the end of a seven to 10-week imposed lockdown and home confinement (from April 29 to May 17, 2020). We found subjective cognitive functioning and mental health severely changed in association with the lockdown. Under government regulations, cognitive complaints were mostly perceived in routine tasks involving attention, temporal orientation and executive functions—with no changes in language abilities. A paradoxical effect was observed for memory, with reduced forgetfulness compared to pre-lockdown. We found higher severity and prevalence of depression, anxiety disorders, abnormal sleep, appetite changes, reduced libido and health anxiety: with mild-to-severe depression and anxiety prevalence climbing to 32 and 36 percent, respectively, under restrictions. Being female, under 45 years, working from home or being underemployed were all identified as relevant risk factors for worsening cognition and mental health. Frequent consumers of COVID-19 mass media information or residents in highly infected communities reported higher depression and anxiety symptoms, particularly hypochondria in the latter. If similar restrictions are reimposed, governments must carefully consider these more vulnerable groups in their decisions, whilst developing effective global and long-term responses to the cognitive and mental health challenges of this type of pandemic; as well as implementing appropriate psychological interventions with specific guidelines: particularly regarding exposure to COVID-19 mass-media reports.

## Introduction

Since the novel coronavirus’s outbreak in December 2019, known as COVID-19, curfews and mass quarantines were adopted worldwide to reduce the pandemic’s impact on healthcare. Italy was the first European country to apply a nationwide lockdown, from March 9 to mid-May, confining people to their homes and to social isolation. Some were allowed out for limited and essential activities, such as for health reasons, food shopping and work.

Psychological and social consequences associated with this stressfully unique situation were expected to be pervasive–affecting mental health, potentially yielding negative long-term effects [1].

Evidence from historical quarantines report massive impacts on mental-health; albeit, those studies were mostly focused on medical staff and virus-affected patients quarantined less than two weeks [2].

Exposure to COVID-19 confinements seems to be an unprecedented condition, incomparable to previous outbreaks, particularly for its global magnitude. These longer-term home confinements still allowed online schooling, some work and socializing—yet, led to depression, anxiety and frustrations due to home-confinement, isolation, disrupted travel, mass-media overload and panic buying [3–5].

The psychological response during the COVID-19 lockdown has been studied since the initial outbreak [5–9]. However, data are conflicting, particularly for the vulnerability factors that can predict public mental health outcomes. Earlier studies yielded mixed interpretations [10], with principal vulnerability factors identified being gender [7, 10, 11], age [5, 7, 12], occupational status [7, 8, 10], exposure to social media and COVID-related information [10, 13]; plus territory of residency, which is related to number of infectious cases [14]. Some earlier research used brief screening scales (e.g., 2-items) to assess depression and anxiety, thus prohibiting the capturing of symptom severities [7], which would be needed for comparing study results [15]. Despite a lockdown’s effect on sleep disorders having been extensively studied [3], only a little evidence on other psychological issues such as eating, sexual disorders and anxiety for health has been reported [7, 16].

Further, up to now, COVID-19’s home confinement impact on subjective cognitive complaints remained an unexplored research topic. A recent review highlighted social isolation has a detrimental effect on cognitive abilities, such as executive functions and memory [17], leading to an unknown scenario about the cognitive consequences related to prolonged periods of social isolations, as imposed by national policy to control the COVID-19 pandemic. Answering this research question seems quite relevant, given cognitive functioning has significant public health consequences. Based on these considerations, the present work explored cognition through subjective cognitive complaints, due to the unfeasibility of performing an objective neuropsychological assessment during the lockdown. Nevertheless, subjective cognitive functioning has been demonstrated to have a significant association with the objective measure of cognitive performance [18], and to significantly predict actual everyday-task functioning [19, 20].

Considering this unprecedented stressful pandemic’s major impact on mental health outcomes [10], in this pioneering report, we address possible changes on subjective cognition as well as mental health related to COVID-19 government restrictions.

To achieve this objective, we administered a nationwide cross-sectional online-survey, during the final phase at seven to 10 weeks into Italy’s lockdown, to reach a large cohort and ensure an adequate representation of Italy’s populace.

First, we explored the changes perceived under restrictions compared to pre-lockdown in subjective cognitive functioning and mental health and we studied their possible inter-relations. We also wanted to determine the prevalence and severity of mental health disorders and related psychological issues such as sleep, appetite, libido and anxiety; comparing pre-lockdown and during lockdown.

Second, based on previous evidence on mental health in the context of COVID-19 lockdown [7, 10, 14], we identified five factors particularly relevant for characterizing potentially vulnerable groups: namely, gender, age, occupational status, COVID-19-media exposure and territory of residency (which is related to number of infectious cases). We explored the influence of these five vulnerability factors on subjective cognitive functioning and mental health outcomes, to identify the most vulnerable groups.

In particular, regarding subjective cognition measures, we focused on possible changes involving attention, executive functions, memory, temporal orientation and language abilities.

Specifically, our hypothesis was: i) the COVID-19 lockdown had detrimental effects not only on mental health, but also on subjective cognitive functioning. We expected individuals to report increased psychological symptoms in sleep, eating, as well as sexual disorders and health anxiety behaviors; ii) based on the vulnerability factors, we expected to identify a set of groups vulnerable to subjective cognitive complaints and mental health disorders.

In our opinion, expanding the knowledge about the lockdown effect on cognitive and psychological domains is crucial to identify potentially vulnerable groups so as to pave a way to implement specific interventions and reduce psychological burdens related to COVID-19 type restrictions. We believe our results can assist government decision-making and healthcare professionals in developing a universal and long-term response to cognitive and mental health challenges of COVID-19 and future pandemics.

## Methods

### Study design and participants

An anonymous online survey was shared through various platforms and mainstream social media from April 29 to May 17, 2020. This timeframe was chosen to assess participants’ responses during the final phase of the COVID-19 pandemic lockdown, imposed by Italy’s parliament from March 9 to May 18, 2020.

To obtain a representative countrywide snapshot of Italy’s populace, which has been differently affected by the pandemic; a snowball sampling method was used. In addition to participants’ own contributions, they were encouraged to share and invite new respondents among their contacts. Of note, we emphasized involvement of the elderly and people with a poor internet access, encouraging participants to help those people complete their surveys. Participation was voluntary and without compensation.

A brief introduction informed participants about our study’s aims. Their informed consent was requested before starting the investigation. The survey took approximately 20 minutes and was anonymous, ensuring data confidentiality.

Responses were considered eligible if participants: i) completed the entire survey, ii) were over 18 years-old and iii) were living in Italy during the pandemic. Among a total of 1559 responses via Qualtrics’ platform, 1242 were classified as eligible according to our inclusion criteria. From this sample, we excluded 27 participants, who reported chronic neurological conditions such as neurodegenerative disease, traumatic brain injury or a history of mental disorders. Our final sample consisted of 1215 participants. This study was conducted in accordance with the Helsinki Declaration and approved by the ethical committee of the School of Psychology University of Padua, and Fondazione Santa Lucia, Rome.

### Survey structure and outcome measures

The survey’s self-reporting questionnaire included three sections. A section collecting sociodemographic features and COVID-19 related information. The other two sections presented two identical self-reporting questionnaires organized into four subsections, wherein participants were asked to think about their condition in normal times (pre-lockdown) versus during lockdown. Specifically, the second section asked about a typical week before the virus’s outbreak (such as during the first week of February—from Monday, 3 to Sunday, February 9, 2020). The third section asked for responses describing their condition during the last week of the lockdown (such as during the last week of April—from Monday April 27 to Sunday May 3, 2020).

Sections two and three were divided into four subsections investigating: i) daily life habits (e.g., average hours spent at home, at workplace or doing sports, number of household residents, drug and psychotropic drug use), ii) subjective memory abilities, iii) subjective global cognitive functioning, and iv) presence of anxiety, depression and other psychological issues, such as sleep disorders, changes in appetite, libido and hypochondria.

#### Sociodemographic, COVID-19 related information and daily life habits

A dedicated questionnaire was set up to collect sociodemographic variables of interest, while the COVID-19 section aimed to collect information on lockdown conditions including job status, living condition, need of psychological consultation, number of times outside home for a walk or shopping and about COVID-19 itself. These included if there had been contraction of COVID-19 infection and its related symptoms, fear, contact with confirmed novel coronavirus carriers and questions about mass-media consumption on COVID-19.

#### Memory functioning

The Prospective and Retrospective Memory Questionnaire (PRMQ) was used to assess memory slips that everyone can make in daily life [21]. PRMQ is a 16-item set, where participants report on a 5-point scale how frequently they experienced some memory mistakes, ranging from ‘very often’ to ‘never’. Higher PRMQ total scores are suggestive of more frequent self-reported memory difficulties. A maximum-minimum total score being 80–16. PRMQ and its subscales have a high reliability as measured by Cronbach’s alpha (for the total score alpha = .89, the prospective scale alpha = .84 and for the retrospective scale alpha = .80).

We slightly modified only Item 12, as it was an unsuitable activity during the lockdown, asking ‘Do you fail to mention or give something to a visitor?’. This item was rephrased as to ‘Do you fail to mention or say something to someone that you had contacted?’.

#### Subjective global cognitive functioning

An ad-hoc 10-item questionnaire was created to assess the subjective global cognitive functioning in performing everyday tasks, identified as feasible activities in a home confinement condition. Items were derived from standardized tools used in clinical practice to assess subjective cognitive complaints: namely, the Perceived Deficits Questionnaire [22] and the Cognitive Change Questionnaire [23], both scales having an adequate reliability with a Cronbach’s alpha of .87 and .94, respectively. Our 10-item questionnaire aimed to assess perceived cognitive problems (by capturing subtle difficulties in performing daily activities, which are related to possible cognitive changes. For example, ‘I have trouble concentrating. For instance, while reading, watching a TV program, working’, ‘I have trouble doing multiple things at once’.

The investigated everyday tasks involved *attention and concentration* abilities (three items referring to difficulties in concentrating on TV programs, talking or looking for an object at home), *executive functions* (three items referring to difficulties in multitasking, problem-solving and decision-making), *temporal orientation* (two items referring to the ability for tracking time, such as remembering the day of the week/month, and an important date such as a birthday), and *language abilities* (two items referring to the ability of finding the proper word and expressing themselves) (for further details see S1 Appendix). Participants were asked on a 5-point scale how frequently they experienced each of the difficulties ranging from ‘very often’ to ‘never’. Total score ranges from 10 to 50, with higher scores representing greater perceived cognitive impairment. A total score as well as subscores for each cognitive domain were computed.

#### Depression and anxiety

The Hospital Anxiety and Depression Scale (HADS) was used to assess presence of anxiety and depression [24]. HADS is a brief 14-item self-rating scale widely used instrument to detect states of anxiety and depression in general practice with excellent psychometric properties [25] and validated in Italian. It has two scales to assess anxiety and depression (HADS-A and HADS-D, respectively), yielding two separate measures of distinct emotional disturbances [26]. A higher total score indicates higher severity in terms of symptoms. To identify presence of clinically significant disturbances, a cutoff score of 8 was adopted. Symptom ranges are 8–10 Mild, 11–14 Moderate, and 15 or higher Severe [25].

#### Sleep, appetite, libido and hypochondria changes

To measure changes in appetite, sleep and interest in sex; we included Beck Depression Inventory (BDI-II) items 16, 18 and 21 [27], which we modified in order to capture changes bidirectionally as behavioral increment or decrement (e.g., increase vs. loss of appetite). Participants were asked to report presence of mild, moderate or significant changes in these dimensions on a 4-point scale, where higher score were suggestive of greater changes. Finally, an item about hypochondria was included. This 4-point scale item of the Hamilton Depression Rating Scale [28] assessed presence of anxiety for health, where a higher score was suggestive of more severe symptoms.

### Statistical analysis

Descriptive analyses were performed for all outcome measures. Preliminary analyses were conducted to check for normality of continuous variables and homogeneity of variance by means of Kolmogorov-Smirnov test and Levene test, with no violations noted (data not shown).

In order to investigate the changes related to COVID-19 lockdown in subjective cognition (i.e., global cognitive functioning and PRMQ) and mental health disorders (i.e., HADS, changes in sleep, appetite, libido and health anxiety), these outcome measures were entered into separate repeated-measure analysis of variance (RM-ANOVA) with Lockdown (pre vs. during-lockdown) as within-subjects factor.

Since depression and anxiety can have an influence on subjective cognitive complaints [18], we verify this relationship through Pearson’s correlations. Pearson’s correlations were run between changes in mental health and cognitive measures, wherein changes scores were computed as score pre-lockdown subtracted from score during lockdown. As this relationship was confirmed, mood and anxiety changes were entered as covariate in RM-ANOVAs, when analyzing cognitive outcomes.

To determine the prevalence of depression and anxiety (HADS-D and HADS-A, respectively), we adopted a cutoff score of 8, which allows the presence of clinically significant disturbances to be identified [25]. We further applied more specific cutoffs to assessed symptoms severity: 8–10 Mild, 11–14 Moderate, and 15 or higher Severe [25]. Percentages of individuals reporting changes in sleep, appetite, libido and hypochondria behaviors were calculated (defined as having a score ≥ 1). Paired categorical variables (pre vs. during-lockdown) were analyzed through McNemar’s test.

To identify potential vulnerable groups to subjective cognitive complaints and mental health, five vulnerability factors were taken into consideration based on previous studies on the effect of the COVID-19 lockdown on mental health [7, 10, 14]. Specifically, the five factors were defined as follow: Gender (male, female); Age (18–25, 26–45, 46–65, >65 years); Working condition (underemployed, working from home, working outside home); COVID-19 mass-media exposure (never, sometimes, often, continuously). Finally, Territory of residency was categorized as a 2-level factor–North versus Center plus South and Islands–due to the different infection rates in Italy, wherein the north regions were the most affected as compared to the others [29].

To investigate the interaction Lockdown ✕ vulnerability factors, cognitive and mental health outcome measures were entered into separate RM-ANOVAs with Lockdown (pre vs. during-lockdown) as within-subjects factor, whilst each vulnerability factor was included as a between-subjects factor. Post hoc analyses were conducted applying the Holm‐Bonferroni correction for multiple comparisons. When necessary, planned contrasts were run to better explore the between-groups rate of change (slope).

In addition, when age, education, depression and anxiety levels differed between-groups, those variables were entered into RM-ANOVAs as covariates, as these variables could have an influence, particularly on subjective cognitive outcomes [18]. Effect sizes were estimated using partial eta squared (ηp^2^) and 95% confidence interval (CI) reported when appropriate. Statistical significance threshold was set at p < .05. We performed statistical analyses using SPSS Statistic, release version 24.0 (Chicago, IL, USA).

## Results

### Sociodemographic and COVID-19 related features

Total sample (*N* = 1215) sociodemographic characteristics as well as COVID-19 related information are shown in Table 1.

**Table 1:** Total sample sociodemographic characteristics and COVID-19-related information.

		Group	n	%
**Age**	mean (SD): 43.18 (14.53)	18–25	119	9.79
		26–45	571	47.00
	min–max: 18–88	46–65	429	35.31
		>65	96	7.90
**Gender**		Female	864	71.11
		Male	351	28.89
**Education**		Middle school	71	5.84
		High school	345	28.40
		Bachelor degree	187	15.39
		Master degree	423	34.81
		PhD/postgraduate	189	15.56
**Territory of residency in Italy**		North	703	57.86
		Center, South and Islands	512	42.14
**Marital status**		Unmarried	580	47.74
		Married	502	41.32
		Separated/divorced	110	9.05
		Widower	23	1.89
**Occupation**		Teacher/researcher	109	8.97
		Medical staff	96	7.90
		Employee	311	25.60
		Freelancer	135	11.11
		Unemployed	49	4.03
		Student	104	8.56
		Retired	124	10.21
		Householder	40	3.29
		Other	247	20.33
**Working condition under COVID-19 lockdown**		Working outside home	297	24.44
		Working from home	535	44.03
		Underemployed	383	31.52
**Fear of COVID-19**		Not at all	87	7.16
		Somewhat	599	49.30
		Moderately	452	37.20
		A lot	77	6.34
**Media exposure about COVID-19 information**		Never	71	5.84
		Sometimes	563	46.34
		Often	438	36.05
		Continuously	143	11.77
**Knowledge COVID-19-infected people**		Family member	95	7.82
		Acquaintance	633	47.90
		Friend	234	19.26
**Knowledge of people died for COVID-19**		Family member	29	2.39
		Acquaintance	309	25.43
		Friend	38	3.13
**Living condition during lockdown**		Alone	157	12.92
		With others	1058	87.08
**COVID-19 infection**		Yes	9	0.74
		No	1206	99.26

*Note*: SD, standard deviation; min, minimum; max, maximum.

### Lockdown effect on subjective cognitive functioning and mental health

As shown in Table 2, under lockdown restrictions, our sample perceived a worsening on global cognitive functioning as compared to normal times (pre-lockdown) (*F*_1,1214_ = 157.34, p < .001, ηp^2^ = .12), but an improvement in memory abilities, as assessed by PRMQ (*F*_1,1214_ = 209.17, p < .001, ηp^2^ = .15) revealing a reduction of everyday-life memory slips, in both prospective and retrospective memory components.

**Table 2:** Subjective cognitive functioning and mental health before vs. during COVID-19 lockdown, in the total sample (*N* = 1215).

	Pre-lockdown	During-lockdown	MD [95% CI]/OR [95% CI]	F/χ2	*p*	η_p_^2^
***Cognitive measures***						
Subjective cognitive complaints	19.99 (5.79)	21.13 (7.45)	-1.14 [-1.43, -0.85]	157.34	< .001	.12
PRMQ (total score)	32.61 (8.90)	30.98 (10.24)	1.63 [1.22, 2.03]	209.17	< .001	.15
Prospective memory	17.45 (4.92)	16.51 (5.71)	0.95 [0.70, 1.19]	181.24	< .001	.13
Retrospective memory	15.16 (4.42)	14.48 (4.98)	0.68 [0.49, 0.88]	177.79	< .001	.13
***Mental health measures***						
HADS-D (*M*, *SD*)	3.83 (3.33)	5.81 (3.97)	-1.98 [-2.19, -1.77]	338.36	< .001	.22
**cutoff** ≥ **8** (*n*)	**15.39%**	**32.30%**	4.25 [3.22, 5.69]	125.73	< .001	
	**(n = 187)**	**(n = 392)**				
mild (8–10)	11.53%	19.03%				
moderate (11–14)	4.03%	10.89%				
severe (≥ 15)	0.32%	2.82%				
HADS-A (*M*, *SD*)	5.21 (3.23)	6.51 (4.03)	-1.29 [-1.5, -1.09]	115.08	< .001	.11
**cutoff** ≥ **8** (*n*)	**21.40%**	**35.72%**	3.00 [2.35, 3.87]	86.00	< .001	
	**(n = 260)**	**(n = 434)**				
mild (8–10)	15.24%	19.19%				
moderate (11–14)	5.48%	12.10%				
severe (≥ 15)	1.21%	4.76%				
Sleep (*M*, *SD*)	0.50 (0.83)	0.94 (0.9)	-0.44 [-0.5, -0.38]	213.52	< .001	.15
% reporting a change (*n*)	33.25%	63.95%	6.57 [5.07, 8.62]	272.95	< .001	
	(n = 404)	(n = 777)				
increase vs. decrease	102/302	360/417				
Appetite (*M*, *SD*)	0.31 (0.67)	0.67 (0.85)	-0.36 [-0.41, -0.31]	212.89	< .001	.15
% reporting a change (*n*)	22.47%	48.15%	7.93 [5.81, 11.07]	240.60	< .001	
	(n = 273)	(n = 585)				
increase vs. decrease	190/83	387/198				
Interest in Sex (*M*, *SD*)	0.39 (0.76)	0.57 (0.84)	-0.18 [-0.22, -0.14]	68.87	< .001	.05
% reporting a change (*n*)	26.01%	38.70%	3.25 [2.47, 4.32]	80.94	< .001	
	(n = 316)	(n = 471)				
increase vs. decrease	72/244	169/302				
Hypochondria (*M*, *SD*)	0.38 (0.71)	0.49 (0.78)	-0.12 [-0.16, -0.08]	35.14	< .001	.03
% reporting a change	26.67%	35.14%	2.81 [2.06, 3.87]	47.95	< .001	
	(n = 324)	(n = 427)				

*Note*: MD, mean difference; CI, confidence interval; PRMQ, Prospective and Retrospective Memory Questionnaire; HADS-D, Hospital Anxiety and Depression Scale for depression; HADS-A, HADS for anxiety; ηp^2^, partial eta squared. Continuous variables were compared by means of repeated-measure analysis of variance, in addition cognitive measures were controlled for depression and anxiety symptoms. Categorical variables were compared through McNemar’s test. Values in bold type indicate the overall prevalence according to HADS cutoff ≥ 8, followed by the specific prevalence according to severity (ranges were defined as: 8–10 mild, 11–14 moderate, 15 and above severe) [25]. The referred changes in sleeping pattern, appetite, libido and hypochondria were defined as having a score ≥ 1. Further, sleep, appetite, libido changes were characterized based on the behavior increase vs. decreased (e.g., increase vs. loss of appetite). Higher total score indicates higher severity in terms of cognitive and mental health symptoms.

Looking at mental health changes (pre- vs. during-lockdown), we found a significant increase of HADS-D and HADS-A scores (as shown in Table 2) suggesting an overall increase in depressive and anxiety disorders related to lockdown. In particular, under restrictions, the prevalence of people reporting clinically relevant depressive symptoms raised up to 32.30% as compared to normal times (15.39%) (χ^2^_1_ = 125.73, p < .001; Table 2), of whom 13.71% and 4.35% showing moderate and severe levels of depression, respectively (χ^2^_1_ = 64.76, p < .001; Table 2).

Similarly, clinically relevant anxiety disorders were present in 35.72% of our sample compared to the 21.40% under normal times (χ^2^_1_ = 86.00, p < .001; Table 2), of whom 16.86% and 6.69% showed moderate and severe levels of anxiety, respectively (χ^2^_1_ = 60.46, p < .001; Table 2).

As reported in Table 2, concerning other psychological issues, we found an increase during the lockdown in problems related to sleep, appetite, libido and hypochondria as compared with pre-lockdown. Changes in sleeping pattern were reported by 63.95% (n = 777), of whom about half experienced insomnia (~54%, n = 417) and the remaining increased sleepiness (~46%, n = 360). For appetite score, the 48.15% (n = 585) reported changes, mostly characterized by an increased appetite (~66%, n = 387). Regarding changes in libido, these were reported by 38.70% (n = 471), mostly characterized by a reduced interest in sex (~64%, n = 302). Finally, during the lockdown, 35.14% (n = 427) showed an increased anxiety for health as compared to the 26.67% before the lockdown.

### Relationship between subjective cognition and mental health changes

**Fig 1:**
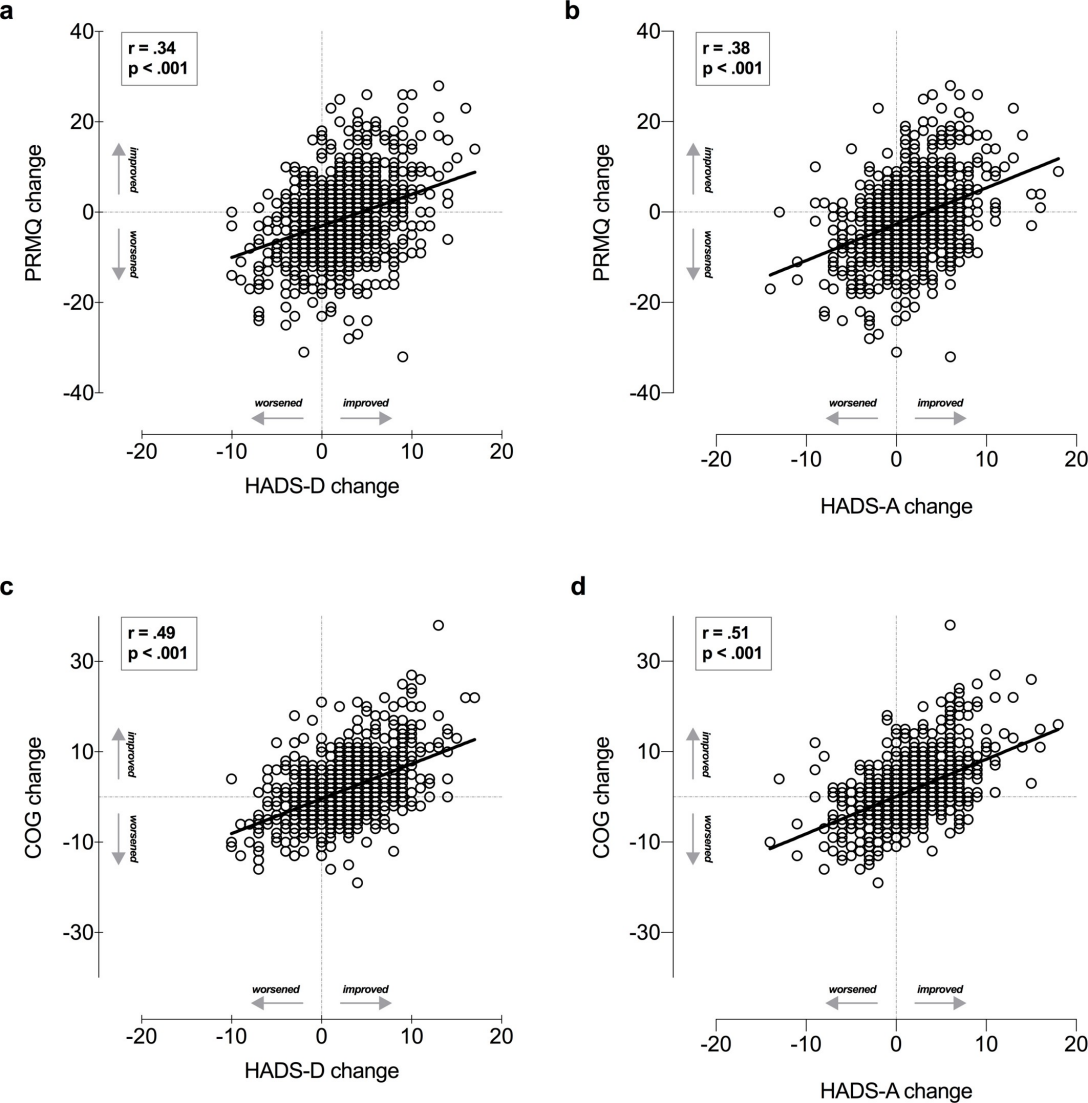
Relationship between subjective cognitive changes and mental health. Positive correlations of depression and anxiety changes with PRMQ (a and b) as well as with subjective cognitive complaints (c and d) scores. HADS-D, Hospital Anxiety and Depression Scale for depression; HADS-A, HADS for anxiety. PRMQ, Prospective and Retrospective Memory Questionnaire. Changes score were computed as score pre-lockdown subtracted from score during lockdown.

In a further analysis of correlations, we found mental health changes were positively associated with subjective cognitive changes. Changes in mood and anxiety were positively correlated with memory domain changes (as assessed by PRMQ) (r = .34; p < .001; r = .38; p < .001 respectively) (Fig 1A and 1B), as well as with subjective cognitive functioning changes (r = .49; p < .001; r = .51; p < .001 respectively) (Fig 1C and 1D). Suggesting depression and anxiety increases were associated with memory and the other cognitive functions worsening, during the lockdown.

### Lockdown and vulnerability factors interaction

Based on previous evidence on the impact of COVID-19 lockdown on mental health [7, 10, 14], five vulnerability factors were identified as particularly relevant: Gender, Age, Working condition during lockdown, Territory of residency (North vs. Center-South-Islands of Italy), and COVID-19 mass-media consumption (i.e., media usage frequency). Thus, to better characterize the most vulnerable groups to experience cognitive complaints and mental issues during COVID-19 lockdown, RM-ANOVAs and post hoc comparisons were run. Results are reported as follow.

#### Gender

**Fig 2:**
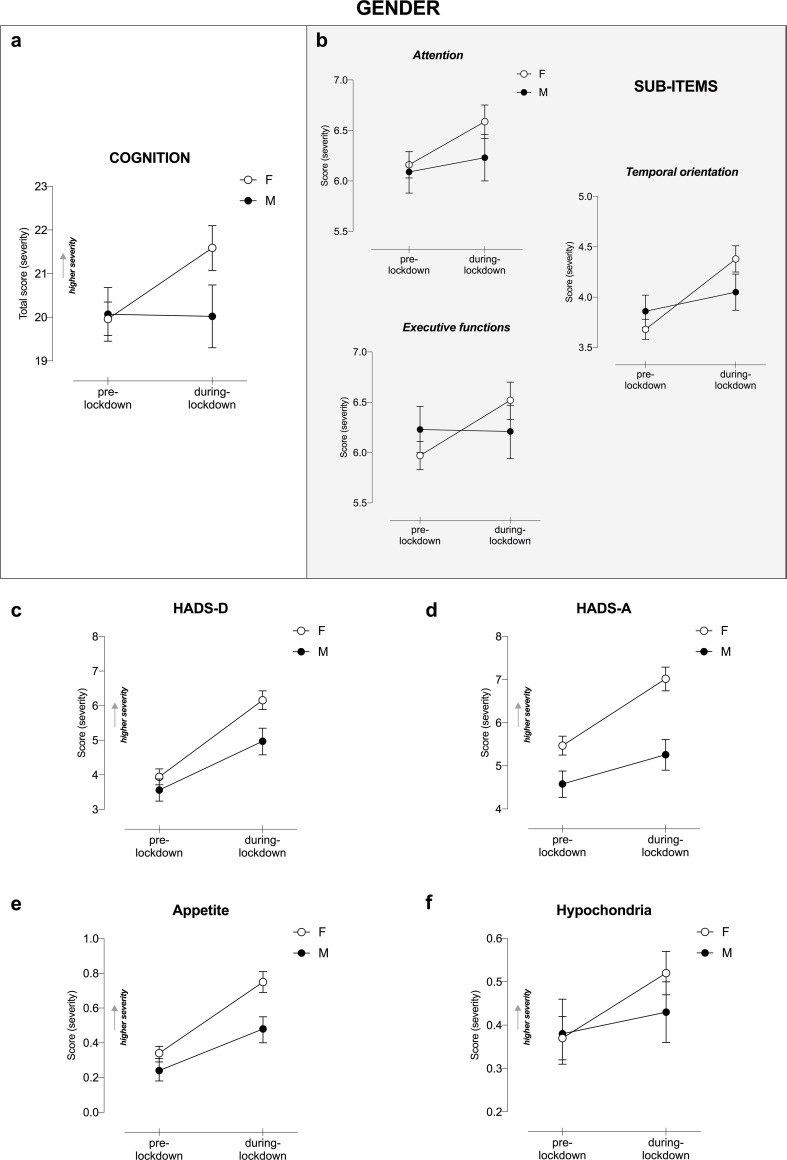
Cognitive and mental health changes as a function of Gender and COVID-19-lockdown. Changes in a) subjective cognitive functioning total score and b) its sub-items, c) HADS-D, d) HADS-A, e) appetite and f) hypochondria as a function of Lockdown and Gender. Only significant interactions Lockdown ✕ Gender are displayed, following RM-ANOVA analysis, entering age as covariate. Higher total score indicates higher severity in terms of cognitive and mental health symptoms. Error bars represent 95% confidence intervals. HADS-D, Hospital Anxiety and Depression Scale for depression; HADS-A, HADS for anxiety.

RM-ANOVA results showed a significant Lockdown ✕ Gender interaction on subjective cognitive functioning (F_1,1211_ = 6.21, p < .013, η_p_^2^ = .005; Fig 2A), revealing women perceived a more pronounced worsening in cognitive functioning as compared to men. In line with this finding, a significant interaction was observed in the sub-items analysis (Fig 2B). As such, women experienced more difficulties in everyday tasks involving attention and concentration abilities (F_1,1211_ = 4.11, p < .043, η_p_^2^ = .003), executive functions (F_1,1211_ = 5.18, p < .023, η_p_^2^ = .004) and temporal orientation (F_1,1211_ = 14.09, p < .001, η_p_^2^ = .011); while no changes were perceived in language abilities (p > .050) during the lockdown.

Regarding mental health, we found a statistically significant Lockdown ✕ Gender interaction in all the investigated outcomes. Lockdown induced a more significant increase in anxiety and depressive symptoms in women than men, as measured by HADS-D and HADS-A respectively (F_1,1212_ = 10.22, p < .001, η_p_^2^ = .008; F_1,1212_ = 13.43, p < .001, η_p_^2^ = .011) (Fig 2C and 2D). As shown in S1 Fig, under restrictions, the prevalence of anxiety disorders increases up to 40.51% in women and 23.93% in men (χ^2^_1_ = 29.88, p < .001; log odds ratio .772 [95% CI = 0.48–1.05]), albeit in the context of a gender difference already present at the pre-lockdown assessment (χ^2^_1_ = 9.63, p < .002). This result suggests that the probability of experiencing depressive disorders was 2.2-fold greater in women than in men during lockdown.

Likewise, we found an interaction between lockdown and gender in depressive disorders, which increased to 35.19% in women; whereas, to 25.07% in men (χ^2^_1_ = 11.68, p < .001; log odds ratio .484 [CI = 0.21–0.76]; S1 Fig); in the context of no differences at baseline (p = .159), suggesting that the probability of experiencing depression during the lockdown was 1.6-fold greater in women than in men.

Furthermore, looking at the other psychological issues related to the lockdown, we found that women experienced a greater change in appetite as compared to men (F_1,1212_ = 8.74, p = .003, η_p_^2^ = .007; Fig 2E), showing increased eating behaviors, and displayed a more pronounced increment in hypochondria and anxiety for health compared to men (F_1,1212_ = 4.50, p = .034, η_p_^2^ = .004; Fig 2F), whereas changes in sleeping patterns and libido were equally affected in both genders.

#### Age

**Fig 3:**
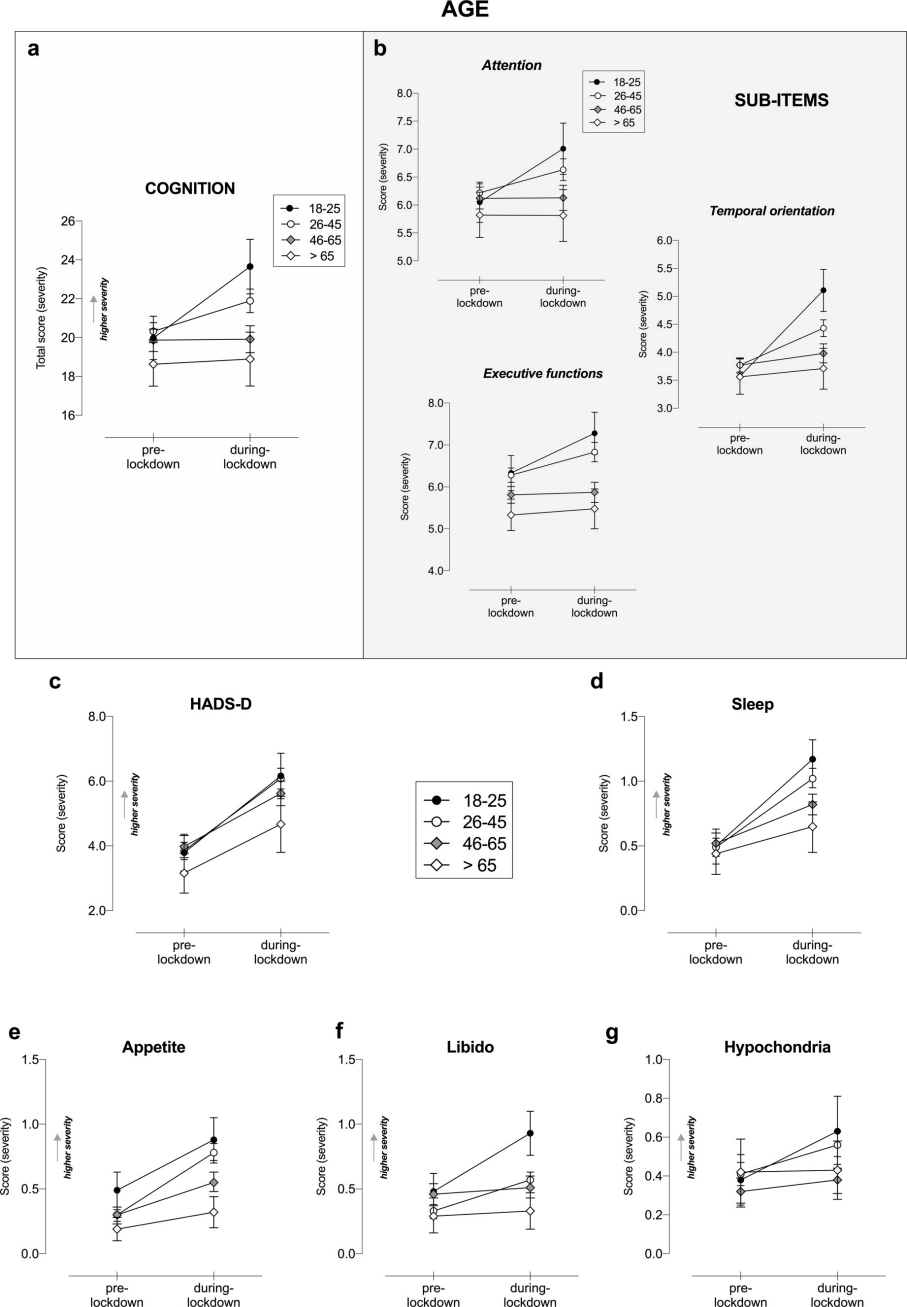
Cognitive and mental health changes as a function of Age and COVID-19-lockdown. Changes in a) subjective cognitive functioning total score and b) its sub-items, c) HADS-D, d) sleep, e) appetite, f) libido and g) hypochondria as a function of Lockdown and Gender. Only significant interactions Lockdown ✕ Age are displayed, following RM-ANOVA analysis, entering education as covariate. Higher total score indicates higher severity in terms of cognitive and mental health symptoms. Error bars represent 95% confidence intervals. HADS-D, Hospital Anxiety and Depression Scale for depression.

Looking at the changes on subjective cognitive functioning during lockdown, we found a Lockdown ✕ Age interaction (F_1,1209_ = 11.65, p < .001, η_p_^2^ = .028; Fig 3A). Post-hoc comparisons revealed that the self-reported worsening of cognitive functioning was statistically significant only in the younger generations: in the 18–25 age group the mean difference (MD) was equal to -3.29 (CI [-4.69, -1.89], p < .001) and in the 26–45 group the MD = -1.34 (CI [-1.99, -0.69], p < .001). Sub-items analyses confirmed this result (Fig 3B), showing the perceived decline was driven particularly by daily life activities involving attention and concentration (F_1,1209_ = 5.50, p < .001, η_p_^2^ = .013; Fig 3B), executive functions (F_1,1209_ = 3.84, p = .009, η_p_^2^ = .009; Fig 3B) and temporal orientation (F_1,1209_ = 21.53, p < .001, η_p_^2^ = .051; Fig 3B), but not language abilities (p > .050). Of note, no interaction was found in the measure related to memory, as assessed by PRMQ.

About mental health, we found a statistically significant Lockdown ✕ Age interaction in HADS-D (F_1,1211_ = 2.93, p < .033, η_p_^2^ = .007; Fig 3C), indicating the lockdown induced a significant increase in depressive symptoms. Since post-hoc results showed mood changes were significant in all age groups (p < .001), to better investigate this interaction, planned comparisons were conducted. Here, we found mood worsening was greater in the 18–26 age group than in the 46–65 group (MD = 0.92, CI [0.19, 1.66], p = .022).

Likewise, we found a Lockdown ✕ Age interaction in sleep (F_1,1211_ = 7.72, p < .001, ηp^2^ = .019; Fig 3D), appetite (F_1,1211_ = 7.43, p < .001, η_p_^2^ = .018; Fig 3E), libido (F_1,1211_ = 11.55, p < .001, η_p_^2^ = .028; Fig 3F) and hypochondria (F_1,1211_ = 4.11, p = .007, η_p_^2^ = .010; Fig 3G). Since post-hoc results showed changes were significant in all age groups (p < .001), to better investigate this interaction, planned comparisons were conducted. These contrasts revealed greater changes in appetite (characterized by an increase) in the group aged 26–45 years as compared to the older generations (45–65 and >65 years) (MD = 0.20, CI [0.09, 0.31], p < .001; MD = 0.33, CI [0.14, 0.51], p < .001, respectively) as well as in the youngest (18–25) group compared to the older (<65) (MD = 0.26, CI [0.03, 0.49], p = .03). Changes in sleep, libido and hypochondria were significantly greater in the younger (<45 years) as compared to the older generations (>45 years). Specifically, sleep changes were greater in 18–25 and 26–45 ages compared to the older groups (p < .010) (18–25 and 26–45 vs. 45–65: MD = 0.38, CI [0.17, 0.59] and MD = 0.21, CI [0.08, 0.34], respectively. While 18–25 and 26–45 vs. >65: MD = 0.47, CI [0.19, 0.75] and MD = 0.30, CI [0.07, 0.53], respectively). Regarding libido, changes were greater in the younger generations compared to the older groups (18–25 and 26–45 vs. 45–65: MD = 0.40, CI [0.24, 0.55], p < .001; MD = 0.20, CI [0.10, 0.30], p < .001, respectively; while 18–25 and 26–45 vs. >65: MD = 0.41, CI [0.21, 0.61], p < .001; MD = 0.22, CI [0.05, 0.38], p < .010, respectively). Similarly, about hypochondria, changes were greater in 18–25 and 26–45 ages compared to the older groups (18–25 and 26–45 vs. 45–65: MD = 0.20, CI [0.06, 0.33], p < .006; MD = 0.11, CI [0.03, 0.20], p < .012, respectively. While 18–25 and 26–45 vs. >65: MD = 0.24, CI [0.06, 0.42], p = .011; MD = 0.16, CI [0.007, 0.31], p = .040, respectively). Interestingly, sleep changes in the younger population were both in terms of poor sleep as well as increased sleepiness. Concerning changes in libido, we found that the youngest individuals (18–25 years) experienced an increased interest in sex, whereas the 26–45 group a reduction under the lockdown.

#### Working condition

**Fig 4:**
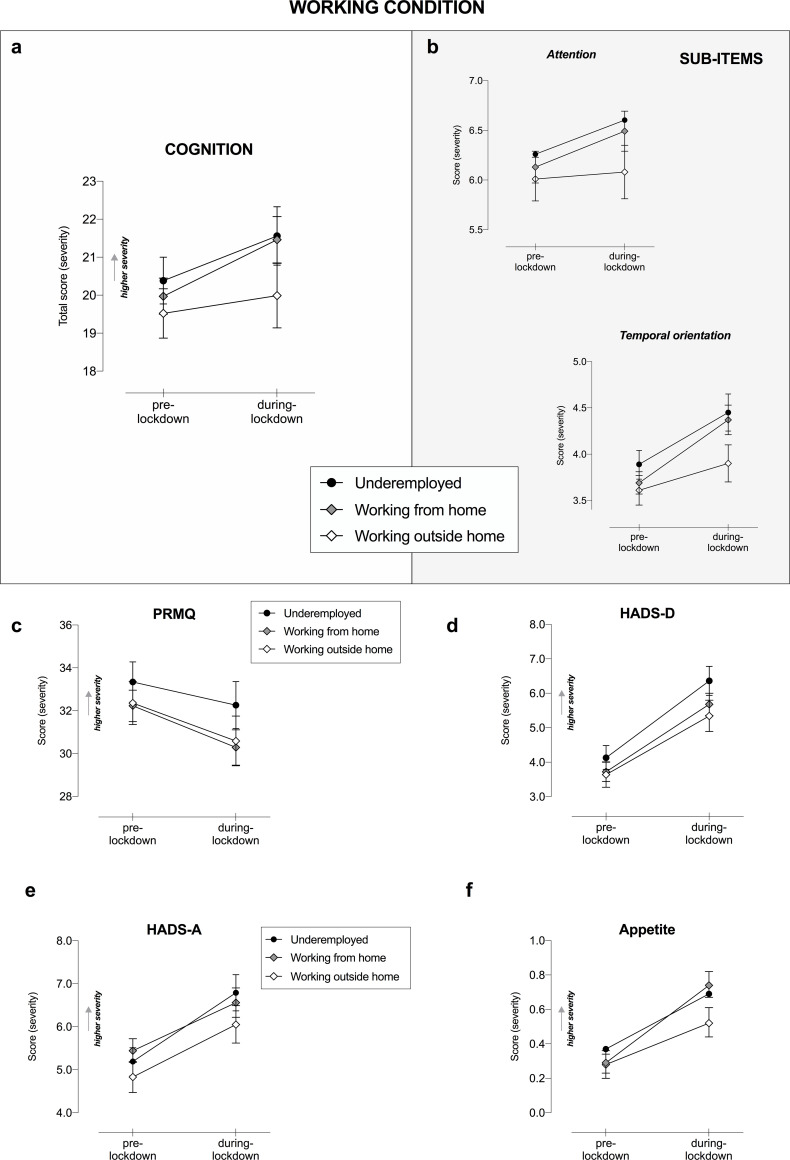
Cognitive and mental health changes as a function of Working condition and COVID-19-lockdown. Changes in a) subjective cognitive functioning total score and b) its sub-items, c) PRMQ, d) HADS-D, e) HADS-A and f) appetite as a function of Lockdown and Working condition. Only significant interactions Lockdown ✕ Working condition are displayed, following RM-ANOVA analysis, entering age and education as covariates. Higher total score indicates higher severity in terms of cognitive and mental health symptoms. Error bars represent 95% confidence intervals. PRMQ, Prospective and Retrospective Memory Questionnaire; HADS-D, Hospital Anxiety and Depression Scale for depression; HADS-A, HADS for anxiety.

A statistically significant Lockdown ✕ Working condition interaction on global cognition (F_1,1209_ = 3.13, p = .040, η_p_^2^ = .005; Fig 4A) was found. Post-hoc analysis following RM-ANOVA revealed the underemployed and working from home groups perceived greater cognitive worsening during the lockdown, as compared to pre-lockdown times (21.56 vs. 20.38; p < .001 and 21.46 vs. 19.97; p < .001, respectively). Aligned with this result, a significant interaction was also observed in the sub-items analysis (Fig 4B), particularly in the attention and concentration domain (F_1,1209_ = 3.52, p = .030, η_p_^2^ = .006) and temporal orientation (F_1,1209_ = 5.38, p = .005, η_p_^2^ = .009).

Regarding memory abilities, we found a statistically significant interaction Lockdown ✕ Working condition effect (F_1,1209_ = 3.02, p = .050, η_p_^2^ = .005; Fig 4C). More specifically, post-hoc analysis highlighted a perceived improvement in memory during the lockdown as compared to normal times in the two working groups (from and outside home) (MD = 2.14, CI [1.14, 3.14], p < .001; MD = 1.76, CI [0.46, 3.06], p < .001, respectively), while no differences were perceived by the underemployed group (MD = 0.81, CI [-0.38, 2.00], p = .321). Noteworthy, perceived improvement in memory domain was positively associated with a positive change of depressive mood and anxiety disorders (Fig 1A and 1B).

The RM-ANOVA on mental health outcomes showed a statistically significant Lockdown ✕ Working condition interaction in HADS-D (F_1,1211_ = 3.35, p = .040, η_p_^2^ = .006; Fig 4D) and HADS-A (F_1,1211_ = 3.99, p = .020, η_p_^2^ = .007; Fig 4E) scales, wherein a significant different impact of lockdown on depressive and anxiety symptoms as a function of the Working condition was observed. As revealed by the post-hoc analysis, however, anxiety and mood changed in all working groups (p < .001). Therefore, to better explore this interaction, planned comparisons were computed. These contrasts revealed the worsening in depressive symptoms was particularly pronounced in the underemployed group, as compared to the two working groups (from and outside home) (MD = 0.58 CI [0.06, 1.11], p = .030; MD = 0.69, CI [0.11, 1.27], p = .020, respectively); similarly, this pattern was observed for anxiety symptoms in underemployed versus working from home comparison (MD = 0.73, CI [0.22, 1.24], p = .005; p = .050). Looking at the changes in the other psychological and habit dimensions, a Lockdown ✕ Working condition interaction in appetite scores (F_1,1211_ = 6.43, p = .002, η_p_^2^ = .011; Fig 4F) was observed. Since post-hoc analysis was unable to capture this difference (the change was significant in all groups), planned comparisons were conducted–revealing the change was greater (in terms of increased appetite) in the working from home group as compared to the underemployed (MD = 0.132, CI [0.019, 0.245], p = .044) and working outside home (MD = 0.214, CI [0.091, 0.336], p = .002) groups. This result however has to be considered with caution.

#### Territory of residency in Italy

**Fig 5:**
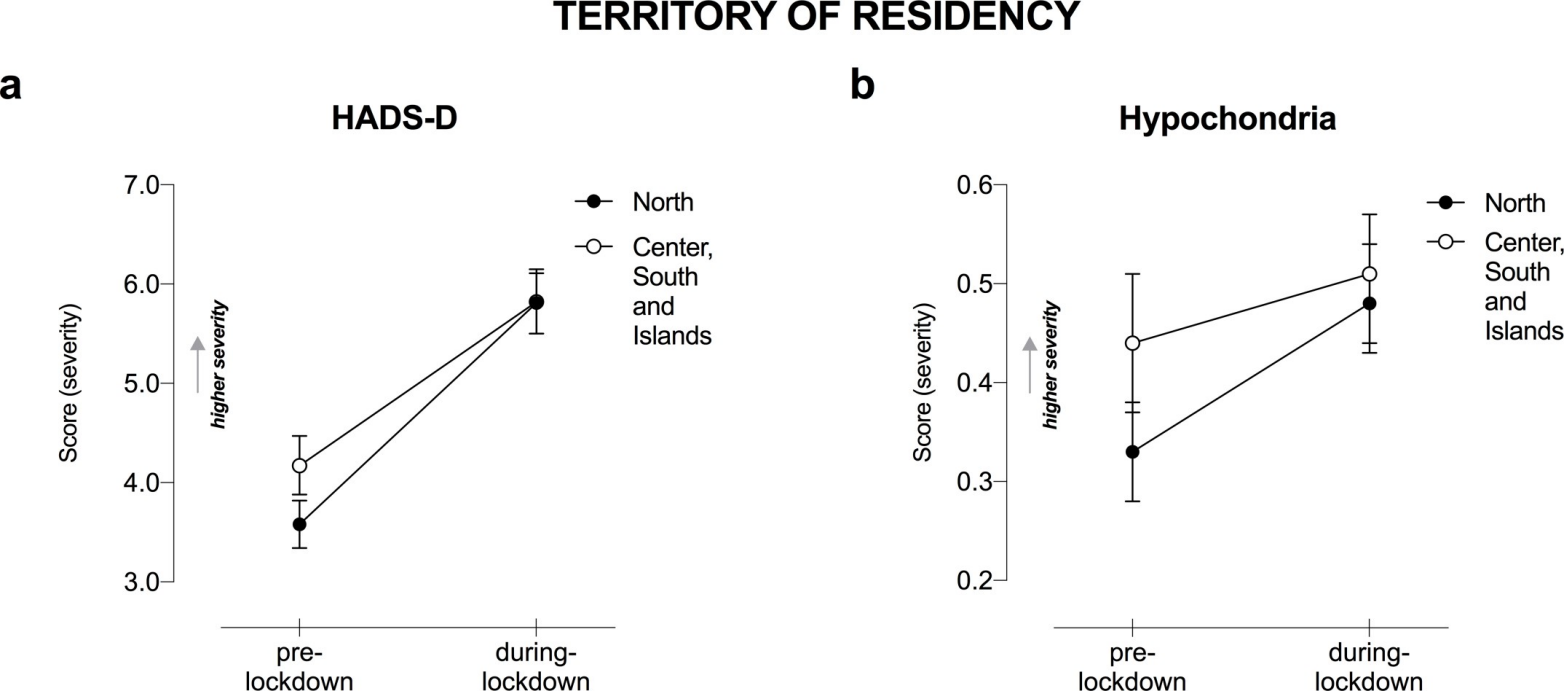
Mental health changes as a function of Territory of residency in Italy and COVID-19-lockdown. Changes in a) HADS-D and b) hypochondria as a function of Lockdown and Territory of residency. Only significant interactions Lockdown ✕ Territory of residency are displayed, following RM-ANOVA analysis, entering age as covariate. Higher total score indicates higher severity in terms of mental health symptoms. Error bars represent 95% confidence intervals. HADS-D, Hospital Anxiety and Depression Scale for depression.

RM-ANOVA results showed a statistically significant Lockdown ✕ Territory of residency interaction on mental health outcomes, in particular on depressive symptoms as measured by HADS-D (F_1,1212_ = 8.48, p = .004, η_p_^2^ = .007; Fig 5A). We found that residents in Northern Italy perceived an increase in depressive disorders as compared to people living in Southern Italy during the lockdown. Likewise, when looking at changes in other psychological issues, an interaction was found in hypochondria associated with the lockdown (F_1,1212_ = 6.41, p = .010, η_p_^2^ = .005; Fig 5B), where again residents in Northern Italy reported greater health-related fears than the other group.

#### Media exposure about COVID-19

**Fig 6:**
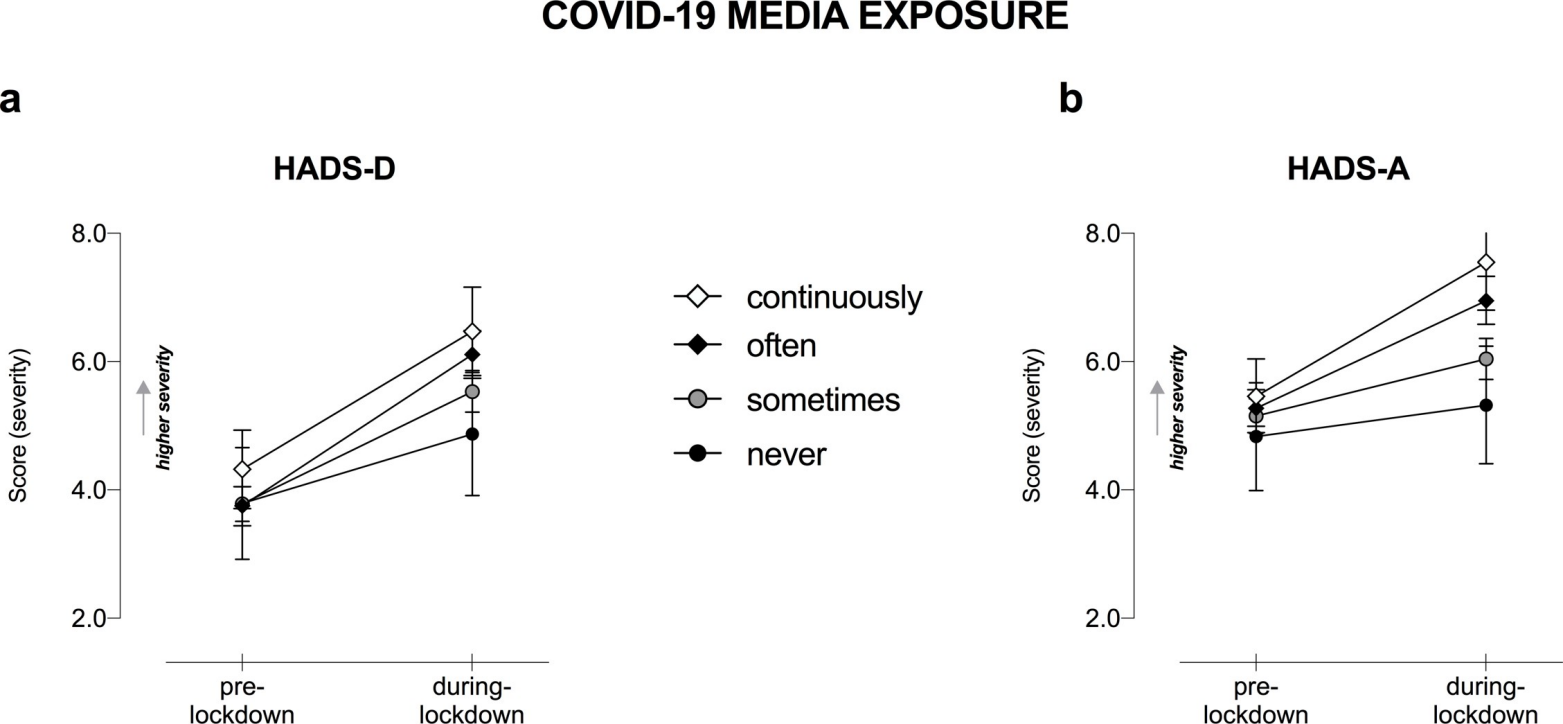
Mental health changes as a function of COVID-19-media exposure and lockdown. Changes in a) HADS-D and b) HADS-A as a function of Lockdown and Working condition. Only significant interactions Lockdown ✕ COVID-19-media exposure are displayed, following RM-ANOVA analysis, entering age as covariate. Higher total score indicates higher severity in terms of cognitive and mental health symptoms. Error bars represent 95% confidence intervals. HADS-D, Hospital Anxiety and Depression Scale for depression; HADS-A, HADS for anxiety.

By categorizing our sample based on the frequency of media consumption about COVID-19 (i.e., media usage frequency) during the lockdown, we found a Lockdown ✕ Media exposure interaction in mental health outcome measures (Fig 6), but not in subjective cognition after including mood and anxiety changes as covariates.

We noted a significant Lockdown ✕ Media exposure interaction in HADS-D (F_1,1210_ = 5.63, p < .001, η_p_^2^ = .014; Fig 6A) and HADS-A (F_1,1210_ = 9.74, p < .001, η_p_^2^ = .024; Fig 6B), in terms of a higher level of depression and anxiety. Since post-hoc results showed mood and anxiety levels significantly changed in all the three groups (Sometimes/ Often/ Continuously) (p < .001), we also conducted the planned comparisons to better investigate this interaction. This analysis revealed mood and anxiety worsening were greater in frequent media-seekers compared to occasional media-seekers (p < .050). Namely, mood worsening was higher in Continuously vs. Never media-seekers (MD = 1.46 CI [0.38–2.54], p = .008) and Often vs. occasional media-seekers (Never and Sometimes): MD = 1.53 CI [0.59–2.48], p = .001 and MD = 0.76 CI [0.29–1.24], p = .002 respectively.

Regarding anxiety, the worsening was greater in frequent media-seekers (Continuously) vs. Never/ Sometimes (MD = 1.91 CI [0.87–2.95], p < .001 and MD = 1.43 CI [0.76–2.11], p < .001 respectively) as well as in frequent media-seekers (Often) vs. Never/Sometimes (MD = 1.38 CI [0.48–2.29], p < .003 and MD = 0.91 CI [0.45–1.36], p < .001 respectively). Whereas changes in the other psychological behaviors were equally affected independently of the COVID-19-media consumption.

## Discussion

Can subjective cognition as well as mental health change in relation to COVID-19-lockdown restrictions? Current worldwide evidence underlines the exposure to this unprecedented stressful condition is increasing the prevalence of mental health disorders, such as depression and anxiety. Our findings corroborate this evidence showing a worsening on mental health disorders, and for the first time our study demonstrates that COVID-19-lockdown has a substantial impact on self-reported cognitive functioning. This study’s results allowed distinct vulnerability groups to be identified that are associated with higher risk of experiencing cognitive worsening and mental health disorders during a pandemic lockdown.

### Perceived cognitive functioning changes

Overall, our study showed Italy’s imposed lockdown restrictions had a detrimental effect on subjective cognitive functioning in its populace. Specifically, we identified the following risk factors of cognitive worsening related to COVID-19 lockdown–female gender, younger age and home confinement (due to teleworking or underemployment conditions). Subjective complaints were mostly perceived in everyday tasks involving attention, temporal orientation and executive functions; while no changes in language abilities were reported.

Conversely, for memory abilities, our findings revealed that working during the lockdown was associated to a perceived memory-domain improvement, both for its prospective and retrospective components, compared to pre-lockdown times. Although, initially, this result appears paradoxical, it is theoretically aligned with the established ‘age-prospective memory paradox’ phenomenon [30], wherein context changes are a key to understanding the memory paradox. Here as well, massive changes to surrounding context and environment under the COVID-19 lockdown are critical to explaining our result. Changes in daily routine during restrictions was characterized by a less frenetic rhythm and a reduction of possibilities, potentially minimizing memory failures. Thus, leading to a subjective improvement of memory.

### Vulnerable groups to subjective cognitive impairment

A recent position paper emphasized an urgent need for collecting high-quality data on mental health, brain function, cognition of COVID-19 lockdown effects across the whole population [1], to identify vulnerable groups and possibly mitigate the psychological consequences of pandemics [2]. Our study extends previous knowledge, highlighting the groups more likely to experience subjective cognitive changes, which have to be considered when implementing specific interventions.

The risk factors we identified converge with those predicting psychological disturbances during the COVID-19 pandemic [6–8, 15] as well as previous isolation/ quarantine conditions [2, 31].

Interestingly, being female was strongly associated with a higher level of cognitive complaints. Despite possible biological vulnerability factors for mental health issues and different stress responses related to gender [32, 33], in this unique context, an explanation could be that women, especially mothers, could become overloaded with family, household and career under restrictions [3, 34]. This resulted in more difficulties managing planned daytime activities due to the intensified career-home relationships, which in turn leads to additional increases in stress, lower self-efficacy and consequently poorer subjective cognitive functioning [35].

Further, we identified being younger and home confinement (due to teleworking or underemployment conditions) as other vulnerability factors related to perceived cognitive worsening. Young adults were identified by earlier studies as being more vulnerable to mental health disorders during the COVID-19 pandemic [11]. Differently from older generations, young adults perhaps experience additional stress due to an uncertain future such as delays in academic career, job insecurity and loneliness. Indeed, profound changes occurred in their social and lifestyle habits leading to longer periods spent online [36]. By contrast, older adults, particularly elderly and retired people while restricted perceived a less pronounced change in their routine and social life, which is generally characterized by less social interactions and more time alone compared to younger people [37].

Job status was also identified as a vulnerability factor. Cognitive worsening was experienced by underemployed individuals or those working from home—but not by working-outside-home employees. This finding suggests ‘confined at home’ workers were more vulnerable and possibly more exposed to financial strain, unemployment fears, work habit changes and reduced work-related social interactions.

As mentioned, sub-items analysis revealed cognitive complaints chiefly involved attentive abilities, executive functions, and temporal orientation. The literature is lacking for quarantine/lockdown effects on cognitive outcomes. Few studies link social isolation with adverse health consequences: including impaired executive functions and faster cognitive decline along with depression, poor sleep, impaired immunity and poorer cardiovascular function [38]. For temporal orientation, our data corroborate earlier findings showing lockdowns induce significant interference when trying to recall the exact day, week, month or hour [40].

By contrast, for memory abilities, we found a paradoxical effect with people experiencing an overall improvement under COVID-19 restrictions. Namely, factors such as working outside or from home were predictive of more effective memory abilities. A functioning characterized by reduced memory slips during routine activities during lockdown. As noted, an explanation can be identified with the salient context changes related lockdown, where employees maintained their schedule but with a less frenetic rhythm and a reduction of possibilities. They had less chance of facing memory slips, leading in turn to a subjective improvement in memory. Conversely, employees stuck at home and essentially jobless, perceived no improvement. Possibly due to their increased stress of pending permanent job loss, financial strain and uncertainty. In this regard, we found subjective memory improvements were moderately associated with lower depressive and anxiety symptoms.

### Mental health changes

A major expected finding of our study was the adverse impact on mental health and psychological behaviors associated with prolonged lockdown. Specifically, higher anxiety and depressive disorder levels were observed; plus negative changes in sleeping pattern, appetite, interest in sex and health-related anxiety.

During the last weeks of Italy’s lockdown, mild/severe levels of depression increased to 32.30% compared to 15.39% pre-lockdown, which is in line with other Italian [8] and Chinese [11] studies suggesting a sharp increase in depression in the general population. This increase was driven by a significant increment of moderate/severe cases, which rose to 13.71% under restrictions as compared to 4.35% pre-lockdown.

For anxiety, under lockdown the prevalence of mild/severe levels of anxiety rose to 35.72% compared to 21.40% pre-lockdown. This corroborates earlier studies of Italy [8] and China [11]. We noted that increased anxiety disturbances under restriction conditions was driven by a significant increase in moderate/severe cases, which rose to 16.86% compared to 6.69% pre-lockdown.

Our results emphasized that under restrictions, increased depressive and anxiety disorders were more than widespread, but also more clinically severe. During the COVID-19 pandemic, monitoring severity seems particularly important as there is an exigency to sooner identify milder clinical cases and implement efficacious intervention, before these evolve to more complex and stable clinical profiles [15].

The lockdown negatively impacted sleep disorders with people experiencing insomnia or increased sleepiness. This type of disorder is extensively reported in the literature [3, 14, 39, 40]. Other relevant psychological issues included: increased appetite, reduced libido and higher level of anxiety for health. This corroborates previous evidence [16, 41–43]. Among these, health anxiety has been demonstrated to be modulated by psychological flexibility [16], while eating more seems to be a typical stress related response [44]. However, combined with confinement and reduced motor activity, it can lead to additional health risks. Taken together, these findings confirmed the COVID-19 pandemic significantly impacted mental health.

### Vulnerable groups to mental health disorders

Among those factors potentially modulating changes in mental health disorders, for both depression and anxiety disorders, we identified distinct vulnerable groups: women, underemployed, and repeated consumers COVID-19-media. In addition, young adults (particularly under 45) and residents in Northern Italy were groups at higher risk for depression.

As stressed by our results and confirmed by other European studies, women have been depicted as more vulnerable to depression and anxiety disorders than men under the COVID-19 lockdown [6, 8]. By contrast, in China an opposite scenario was reported [5]. Our study highlighted an important gender difference associated with lockdown. It showed the probability of developing depression was 1.6-fold and for anxiety 2.2-fold greater in women than men; in the context of a pre-lockdown parity for depression between genders and only a slight difference for anxiety. Differently from men, during the lockdown, women experienced a change in terms of appetite, which increased for the majority of our sample. Women also reported higher health-related anxiety levels than pre-lockdown. However, we found no differences between genders in terms of changes in libido and sleep disturbances, differently from previous study [7]. Despite the established gender gap showing higher prevalence of mental health disorders in women [33], our data underlines the severity of lockdown on psychological well-being, with women being more vulnerable.

Young adults resulted as being at higher risk for depression and psychological issues than people over 45. Namely, our younger groups below 45 were more likely to experience sleeping disorders, increased-eating behaviors, changes in sexual desire and increased hypochondria. Although it is a counterintuitive result as the COVID-19 fatality rate is higher among the elderly [45]. Our findings are in line with other evidence identifying young adults as vulnerable for psychological issues associated to the COVID-19 lockdown [7, 11, 46]. Youth are exposed to uncertainty for prolonged school-closings, precarious employments, social and lifestyle upheavals; with more time spent on social networking–aspects, easily triggering stress leading to mental health issues [5, 37, 47].

As expected, being underemployed was associated with increased depression and anxiety. Our sample yielded a reduction of about 1.5 working hours per day [6.75 (3.37) vs. 5.03 (3.78), F_1,1214_ = 329.92, p < .001, η_p_^2^ = .214].

Differently from previous evidence, we did not find workers employed during lockdown, which includes healthcare professionals, to be at a higher risk for mental illness [7, 13, 15, 48], particularly with a high level of burnout [48]. We surmised this is due to the low representation of front-line healthcare workers in our sample (only 7.9%), with the majority of regular workers still working and not reporting troubles or financial insecurity compared to our underemployed group [8, 49].

Another risk factor has been identified in the Italian region of residence. Northern Italy during the COVID-19 pandemic revealed increased mental health disturbances, which included more depression and hypochondria. The north was considered as Italy’s ground-zero–with its highest infection and death tallies. Our results are consistent with previous pandemics evidence such as for SARS, showing residents of the most densely infected regions having a higher risk for developing mental health disorders, particularly depression [50]. This increased anxiety-for-health level is possibly explained as people in high infection-prevalence areas perceive themselves as more vulnerable to the disorder [14].

Importantly, among groups more vulnerable to depression and anxiety, we identified repeated seekers of COVID-19-media. Our results agree with a recent study from Spain showing elevated anxiety, but not depression, in association to increased time following COVID-19 news [51]. Of note, anxiety and uncertainty can lead to further media consumption and additional distress, generating a vicious cycle [52]. Staying on the internet longer during lockdown has been linked with depression disorders [7]. Since overexposure to COVID-19-media amplifies distress, leading to mental illness; future research and guidelines are needed to promote wellbeing by prescribing optimal patterns of media consumption through a pandemic [47].

### Relationship between subjective cognitive and mental health changes

In line with previous evidence [18], we found a strong association between subjective cognitive complaints and depressive or anxiety disorders: as the psychological symptoms increase, the daily cognitive performance was impaired. These findings stressed the importance of considering perceived cognitive functioning in the context of mood and anxiety-related symptoms, as it is for an objective cognitive assessment. Of note, the frequent exposure to COVID-19 mass-media did not result as being a vulnerability factor for cognitive complaints after controlling for the confounding effect of mental health changes. Hence, we surmise frequent seekers of COVID-19 information tended to perceive more subjective cognitive complaints due to anxiety/depression disorders rather than an actual worsening in cognition.

### Limitations

There are a few shortcomings and limitations in this work to be considered. Our survey sampling was based on the snowball method, involving an online invitation, but leaving unexplored the population not using networked devices. Considering our survey’s online distribution, no data about non-participants were collected and no refusal rate was registered. However, during our own home confinement, this was our only practical feasible sampling method. For a heterogeneous sample, we encouraged participants to invite the elderly and people with a poor internet. Our sample seems to have an adequate representation of Italy with an age range 18 to 88, years, a pan-Italy distribution and diverse educational levels. It is less balanced for gender, as about 70% were female. Subjective complaints and mental health outcomes are based on self-reported measures rather than clinical diagnoses, although most selected scales were validated [21, 27] or derived from standardized tools [22]. Regarding cognition, we used self-reported measures to assess cognitive functioning, we are aware that it could have been objectively assessed using online neuropsychological testing, which seems a promising avenue for research methodology [53], albeit its clinical validity still needs to be confirmed [54]. Here, mostly due to the lockdown restrictions, it was unfeasible to implement such a complex study design. Further, our rationale was based on previous studies demonstrating subjective complaints significantly predict the actual everyday-task functioning [19, 20], emphasizing their strong validity and potential clinical value [55]. However, asking participants to self-evaluate their cognitive functioning can lead to self-judgment biases, particularly in those individuals with anxiety/mood disorders, self-awareness and/or memory deficits. Here, memory deficits could have interfered with their own retrospective evaluation. Although, in the current research to partially avoid this issue, we excluded individuals with neurodegenerative disorders. Of note, adopting online experiments to assess objectively the psychological functioning has been demonstrated to be a valuable approach, particularly under social restrictions [56]; future studies, evaluating cognition during pandemics, have to take into consideration this approach in their study design. In this regard, a recent review by Marra and colleagues (2020) paved the ground to implement neuropsychological assessments via telehealth (i.e., teleneuropsychology) confirming the validity of this tool to assess cognitive functioning in older adults [57].

Depression and anxiety prevalence were based on HADS, which has excellent psychometric properties and is extensively used in the general clinical practice [25]. Finally, although our study was not designed as longitudinal, it comprised a 2-time points assessment (pre-lockdown vs. lockdown), yielding a direct comparison of the self-reported measures within the same subject; thereby minimizing possible variability issues. Hence, differently from previous evidence on mental health [15], we are able to infer that observed changes were possibly due the COVID-19- lockdown effect.

## Conclusions

This is one of the earlier reports showing lockdown can severely impact subjective cognitive functioning, along with mental health disorders. We showed that while obligated to physical and lifestyle restrictions, people can experience subjective cognitive complaints in attentive, executive, temporal orientation abilities. All these while paradoxically perceiving an improvement in memory domain, with a reduced forgetfulness in daily activities. We found higher severity and prevalence of depression, anxiety disorders and other psychological issues involving sleep, appetite, libido and hypochondria.

Italy’s parliament imposed new curfews, domestic and international travel restrictions, reduced store hours, shorter restaurant opening hours, limits to personal mobility outside the home and similar starting on November 6, 2020 as part of its COVID-19 pandemic experience. Knowing the associated cognitive and psychological consequences are crucial as they are fundamental for providing effective and supportive psychological interventions, particularly to vulnerable populations. Because, there is no assurance these government interventions will not happen again.

Our results characterized those being female, under 45, underemployed, residents in high infection-prevalence areas and exposed to COVID-19 related mass media reports as being particularly vulnerable.

We believe future researches are required to define the long-term consequences of epidemic lockdowns on subjective cognition and mental health disorders, as well as for defining specific guidelines, especially for COVID-19-media consumption, to minimize the psychological impacts.

## Supporting information

S1 Fig Prevalence of mild depression and anxiety according to gender, pre- and during-lockdown. Mild symptoms assessed with a cutoff ≥ 8 of a) HADS-D and b) HADS-A scales. HADS-D, Hospital Anxiety and Depression Scale for depression; HADS-A, HADS for anxiety.
(TIF)

S1 Appendix (PDF)
