# Supplementary figures and images for: Cognitive and mental health changes and their vulnerability factors related to COVID-19 lockdown in Italy
Source: PLoS One. 2021 Jan 27;16(1):e0246204. doi: 10.1371/journal.pone.0246204 (PMC7840042; doi:10.1371/journal.pone.0246204)

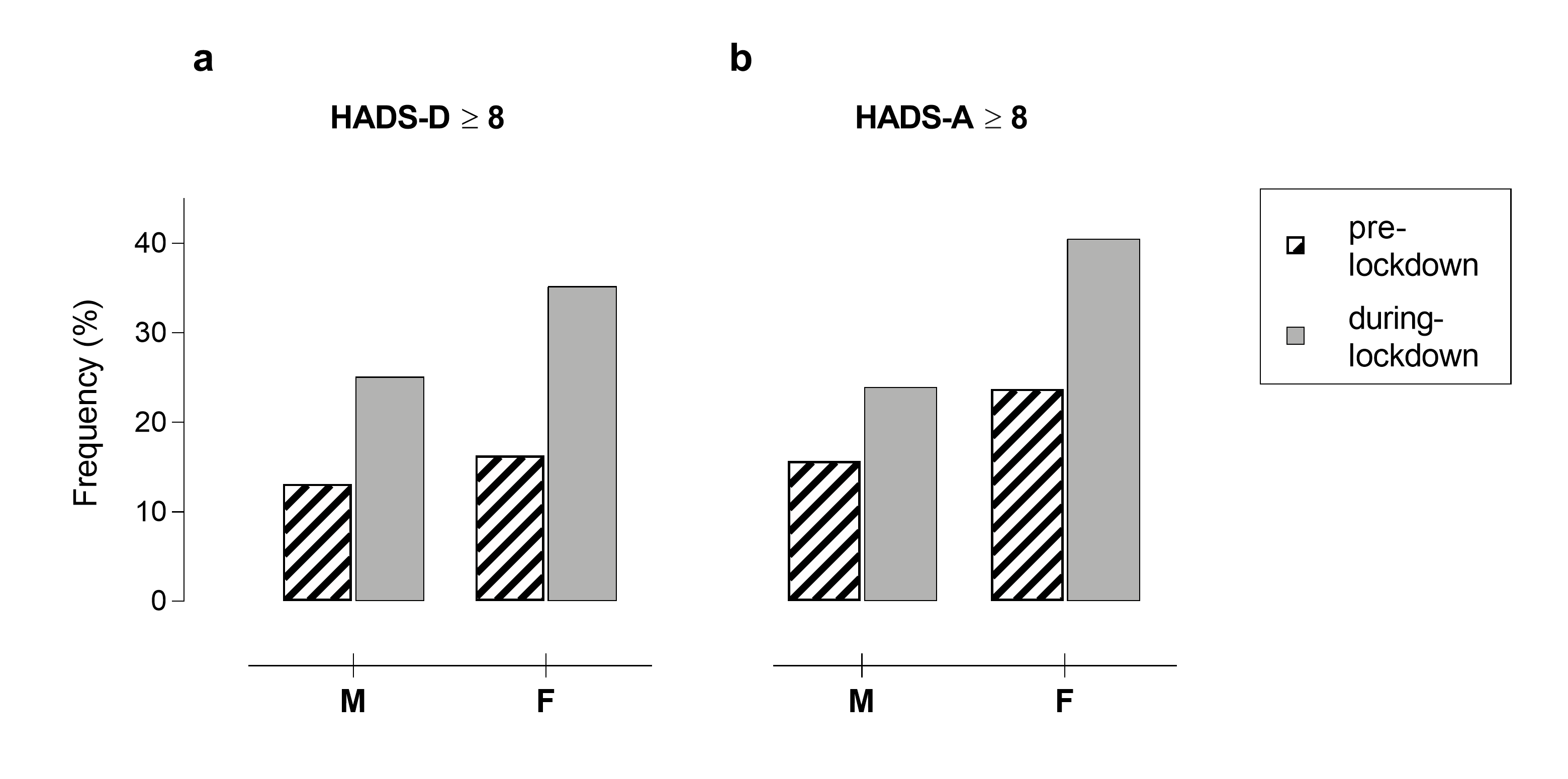

Supplement: S1 Fig — Mild symptoms assessed with a cutoff ≥ 8 of a) HADS-D and b) HADS-A scales. HADS-D, Hospital Anxiety and Depression Scale for depression; HADS-A, HADS for anxiety. (TIF) [file pone.0246204.s001.tif]
