# Supplementary material for: Cognitive and mental health changes and their vulnerability factors related to COVID-19 lockdown in Italy
Source: PLoS One. 2021 Jan 27;16(1):e0246204. doi: 10.1371/journal.pone.0246204 (PMC7840042; doi:10.1371/journal.pone.0246204)
Supplement: S1 Appendix — (PDF) [file pone.0246204.s002.pdf]

S1 APPENDIX

Questionnaire to assess global cognitive functioning and items categorization according to the involved cognitive functions

INSTRUCTIONS: Please answer all questions as accurately as possible, indicating how often the following situations occurred to you (very often, quite often, sometimes, rarely, never).

	very often	quite often	sometimes	rarely	never
1. Have you had trouble concentrating (for instance, while reading, watching a TV program, working)?					
2. Have you had trouble doing multiple things at once?					
3. Did you experience greater reasoning or judgment difficulties (such as making an inappropriate purchase, making wrong financial decisions)?					
4. Did you forget the day of the week, the date or the correct year?					
5. Did you forget an important date (like an acquaintance's birthday, an anniversary)?					
6. Have you had trouble completing something you started (for instance, interrupting a task to respond to messages or emails, although not urgent)?					
7. Have you had trouble to express your opinion, for instance by losing the thread of the speech of what you were saying?					
8. Have you had trouble finding the right word, having the feeling of having it "on the tip of the tongue"?					
9. Have you had trouble telling another person what you had just seen or heard?					
10. Have you had trouble remembering where an object was usually kept at home (for instance, you forgot where to find a tool in the kitchen or in the garage)?					

**Cognitive functions**	**Items**
Attention and concentration	1, 7,10
Executive functions	2, 3, 6
Temporal orientation	4, 5
Language	8, 9
